# Food Security and Leukocyte Telomere Length in Adult Americans

**DOI:** 10.1155/2017/5427657

**Published:** 2017-08-29

**Authors:** Mohsen Mazidi, Andre Pascal Kengne, Hassan Vatanparast

**Affiliations:** ^1^Key State Laboratory of Molecular Developmental Biology, Institute of Genetics and Developmental Biology, Chinese Academy of Sciences, Beijing 100101, China; ^2^Institute of Genetics and Developmental Biology, International College, The University of Chinese Academy of Science, Beijing 100101, China; ^3^Non-Communicable Disease Research Unit, South African Medical Research Council, Cape Town, South Africa; ^4^Department of Medicine, University of Cape Town, Cape Town, South Africa; ^5^College of Pharmacy and Nutrition, University of Saskatchewan, 104 Clinic Drive, Saskatoon, SK, Canada S7N 2Z4

## Abstract

**Background and Purpose:**

Leukocyte telomere length (LTL) is a biomarker of biologic age. Whether food security status modulates LTL is still unknown. We investigated the association between food security and LTL in participants of the 1999–2002 US National Health and Nutrition Examination Survey (NHANES).

**Methods:**

Analysis of covariance (ANCOVA) was used to evaluate the association between food security categories and LTL controlling for sex, race, and education and accounting for the survey design and sample weights.

**Results:**

We included 10,888 participants with 5228 (48.0%) being men. They were aged on average 44.1 years. In all, 2362 (21.7%) had less than high school, 2787 (25.6%) had achieved high school, while 5705 (52.5%) had done more than high school. In sex-, race-, and education-adjusted ANCOVA, average LTL (T/S ratio) for participants with high food security versus those with marginal, low, or very low food security was 1.32 versus 1.20 for the age group 25–35 years and 1.26 versus 1.11 for the 35–45 years, (*p* < 0.001).

**Conclusion:**

The association between food insecurity and LTL shortening in young adults suggest that some of the future effects of food insecurity on chronic disease risk in this population could be mediated by telomere shortening.

## 1. Introduction

Food security is defined in the State of Food Insecurity in the World 2001 as a situation that “exists when all people, at all times, have physical, social and economic access to sufficient, safe and nutritious food that meets their dietary needs and food preferences for an active and healthy life” [1]. There is an emerging evidence suggesting an association between food security and chronic disease occurrences such as cardiovascular and diabetes [2–5]. Telomeres are guanine-rich sequence (TTAGGG) that play active role in the control of cellular lifespan and chromosome integrity in eukaryote cells [6]. Accelerated telomere shortening might be the consequence of increased exposure to oxidative stress and inflammation [7]. Available evidence suggests that short leukocyte telomere length (LTL) is linked with an increased risk for various noncommunicable diseases [8], as well as aging.

Studies have reported an effect of diet composition on DNA telomere length (TL) [6]. Mediterranean diet which is made up predominantly of vegetables, fruits, grains, fish, monounsaturated fatty acids, and dairy products has been reported to have a protective effect on telomere length [9], while diet rich in monounsaturated fatty acids, through inflammation promoting effect, may accelerate telomere shortening [10]. However, the association between food security and telomere length has yet to be explored. Food security as assessed in national surveys can be considered as a practical tool to evaluate food availability in relation with diseases risk. Hence, we aimed to determine the association between food security and LTL among adult Americans who participated in the Health and Nutrition Examination Survey (NHANES) during 1999 to 2002.

## 2. Methods

In NHANES cross-sectional surveys, conducted periodically by the US National Centre for Health Statistics (NCHS), participants are visited at home by trained workers to collect data on their demographic characteristics and behaviours, by administering a survey questionnaire [11]. Then mobile examination units are used to conduct physical and paraclinical examination, as well as collecting biological specimens for laboratory analyses. NHANES' protocol is approved by the NCHS Research Ethics Review Board, and informed consent was obtained from all the participants [12, 13]. LTL was measured from purified DNA isolated from whole blood, using the Puregene (D-50 K) kit protocol (Gentra Systems, Inc., Minneapolis, Minnesota). The quantitative polymerase chain reaction method was then used to measure LTL relative to standard reference DNA [also known as the telomere-to-single copy gene (T/S) ratio] [13–15]. A validated questionnaire developed by the US Department of Agriculture (USDA) was used to assess income-related household food security over the prior 12 months [16]. Using validated cut points, we categorized participants as (a) adult full food security (no affirmative response in any of these items), (b) adult marginal food security (1-2 affirmative responses), (c) adult low food security (3–5 affirmative responses), and (d) adult very low food security (6–10 affirmative responses) [16]. For data analysis, we followed the guidelines defined by the Centre for Disease Control and Prevention for NHANES datasets, taking into consideration the masked variance and weighting scheme [13, 17]. Sex-, education-, and the race-adjusted mean LTL was compared across categories of food security (high food security versus marginal, low, or very low food security) using analysis of covariance (ANCOVA). All tests were two-sided, and statistically significant results were considered at *p* < 0.05.

## 3. Results

The 10,888 participants included 5226 (48.0%) men, 7665 (70.4%) non-Hispanic Whites, 1186 (10.9%) non-Hispanic Blacks, 751 (6.9%) Mexican-Americans, 775 (7.1%) other Hispanic ethnicity, and 511 (4.7%) participants from other ethnic groups (Table 1). The mean age was 47.0 (standard error of the mean = 0.3), with a marginal difference between men and women (47.5 versus 46.6 years, *p* = 0.069). Overall, 2362 (21.7%) participants had less than high school education, 2787 (25.6%) had high school education, and 5705 (52.5%) had more than high school education. Sex-, race-, and education-adjusted mean LTL (T/S ratio) was longer in participants with high food security compared to those with low food security (marginal + low + very low) just in two age categories: [25–35] years (1.32 versus 1.20) and [35–45] years (1.26 versus 1.11), both *p* < 0.001 (Table 2). There was no significant difference within other age categories (all *p* > 0.05).

## 4. Discussion

Participants with higher food security status in the current study, particularly at their younger age, had longer LTL. Epidemiological studies have reported an inverse association between dietary vitamin and mineral consumption with oxidative stress [18], which suggests that dietary micronutrients may exert protective effects on telomere length through antioxidative effects. It has been proposed that the high cost of nutrient dense healthy foods, particularly lean meats, milk, and dairy products, may limit the ability of the poorer segments of the population to consume them [2]. For instance, it has been reported that food insecurity could trigger the intake of low-cost high-energy dense foods, which also tend to have high sodium content [19, 20]. Food insecurity has also been associated with lower quantity and less variety of fruits and vegetable (main source of several phytochemicals including *α*-tocopherol, retinol and *β*-carotene, and vitamins) consumption [21]. We can speculate that our findings reflect the reverse association between lower food security and higher intake of the fruit and vegetable (the main source of the vitamins, minerals, and antioxidant in diet) [21]. A recent study in Japan also reported a positive association between buccal TL and daily intake of antioxidant vitamins including lycopene, phenolics, favonoids, resveratrol, phloridzin, and pectin [22]. In the same line, we have recently reported that participants with higher intake of mineral and vitamin consumption have longer TL [23]. In most Western countries, including US and Canada, fresh fruits and vegetables as the indicators of quality food are not accessible to low-income families [24–26].

Food insecure individuals may not benefit from the anti-inflammatory properties of antioxidants due to low consumption of their food sources. Several potential mechanisms have been proposed for a positive association between antioxidant levels and TL. In vitro and in vivo studies have reported that lutein, zeaxanthin, and vitamin C prevent DNA breakage and modulate DNA repair through their antioxidative activity [27, 28]. Other proposed mechanisms, particularly for the protective effect of lutein, zeaxanthin, and vitamin C, include their effects on immune modulation, anti-inflammatory activity, modulation of apoptosis, and lymphocyte proliferation [28, 29].

The link between chronic disease and food security status has been recently reported [2]. Life course stage, chronic stress, and food environment are considered as the main moderators of metabolic disturbance leading to chronic diseases [24]. The quality of food and stress from lack of food and hunger within the household can trigger a cascade of stress hormones and create a metabolic imbalance, ultimately resulting in chronic metabolic diseases such as metabolic syndrome and diabetes. The association between food security and TL may, in part, explain why food insecure individuals are more prone to chronic diseases.

The findings from the current study should be considered in the context of some limitations. The cross-sectional nature does not allow inference about causality. We did not have data on repeated measures of TL in the same participants over time to elucidate temporality of these findings.

The link between food security and LTL in young participants in our sample may indicate the potential of this biomarker in predicting the future occurrence of nutrition-related noncommunicable diseases. Many people build up chronic disease risk during childhood and early adulthood. The observed associations between food security and LTL strengthen the growing evidence that food insecurity may be a determinant of poor overall health and propensity to chronic diseases considering the importance of telomere length maintenance in preventing noncommunicable diseases.

## Figures and Tables

**Table 1:** Sample size and weighted characteristics of NHANES 1999–2002 adult participants.

		ALL	
		N	Weighted distributions of the participants
Sex	Men	5226	48.0%
	Women	5661	52.0%

Age (years), Mean ± SEM		10,888	44.1 ± 0.3

Race/ethnicity	White (non-Hispanic)	7665	70.4%
	Non-Hispanic Black	1186	10.9%
	Mexican-American	751	6.9%
	Other Hispanic	773	7.1%
	Other	511	4.7%

Education level	Less than high school	2362	21.7%
	Completed high school	2787	25.6%
	More than high school	5705	52.5%

NHANES: National Health and Nutrition Examination Survey.

**Table 2:** Adjusted (sex, race, and education) mean of TL for different categories of food security across the different ages.

Age categories	Food security categories		*P* value
	High (*n* = 8643)	Marginal + low + very low(*n* = 2245)	
18–24.9 (*n* = 2070)	1.41	1.39	0.235
25–34.9 (*n* = 1783)	1.32	1.20	<0.001
35–44.9 (*n* = 1752)	1.26	1.11	<0.001
45–54.9 (*n* = 1540)	1.04	1.01	0.328
55–64.9 (*n* = 1387)	0.98	0.95	0.694
Upper than 65 (*n* = 2355)	0.87	0.88	0.835

TL: telomere length (T/S ratio), value expressed as a mean and SEM. Results from the analyses of covariance statistical procedure.
